# Urinary Incontinence among Pregnant Women in Third Trimester of Pregnancy in a Tertiary Care Center: A Descriptive Cross-sectional Study

**DOI:** 10.31729/jnma.6914

**Published:** 2021-08-31

**Authors:** Atit Poudel, Ganesh Dangal, Madhu Shrestha

**Affiliations:** 1 Department of Obstetrics and Gynaecology, Paropakar Maternity and Women's Hospital, Thapathali, Kathmandu, Nepal; 2 Department of Obstetrics and Gynaecology, Kathmandu Model Hospital, Exhibition Road, Kathmandu, Nepal

**Keywords:** *pregnancy*, *third trimester*, *urinary incontinence*

## Abstract

**Introduction:**

Urinary incontinence is an involuntary passage of urine. The aim of the study was to find the prevalence of urinary incontinence among pregnant women in the third trimester of pregnancy at a tertiary care center.

**Methods:**

This descriptive cross-sectional study was conducted in a tertiary care center from March 2021 to May 2021. Ethical approval was obtained from the Institutional Review Board (reference number: 854/2077/78). Convenience sampling method was used. A descriptive analysis of socio-demographic profile and urinary incontinence symptoms were recorded on International Consultation on Incontinence Questionnaire-Urinary Incontinence-Short Form questionnaire and analysis were done using Statistical Package for Social Sciences 27. Point estimate at 95% Confidence Interval was calculated along with frequency and proportion for binary data.

**Results:**

Among 277 pregnant women admitted in the antenatal ward, urinary incontinence was present in 26 (9.4%) (95% Confidence Interval= 5.96-12.84). Among them stress urinary incontinence 16 (61%) was most common followed by mixed incontinence 6 (23%). Majority of them 18 (69.3%) had small leaks with almost all 25 (96.2%) having only a mild to moderate impact on the quality of life. Majority 197 (71.2%) had features of lower urinary tract syndrome.

**Conclusions:**

Our study showed similar prevalence of urinary incontinence compared to other international studies.

## INTRODUCTION

Urinary incontinence (UI) is any involuntary passage of urine.^1^ This is a common problem with prevalence varying between 6-75%, with an average of 41% and worsen during pregnancy.^2^ The most common type of UI in pregnancy is Stress urinary incontinence followed by Urgency urinary incontinence and Mixed urinary incontinence.^2-4^

The age of pregnant mother, parity, smoking, alcohol, coffee intake, BMI, physical activity, prior history of UI, urinary tract infection (UTI), and constipation are common risk factors associated with UI in pregnancy.^5^ Many women suffer from UI in silence due to social stigma and embarrassments placing their social and economic lives in jeopardy.^6-9^ In Nepal, the urinary incontinence in pregnancy is unaddressed.^10^

The aim of this study was to find prevalence of urinary incontinence among pregnant women in the third trimester of pregnancy in a tertiary care hospital.

## METHODS

This is a descriptive cross-sectional study conducted among pregnant women at Paropakar Maternity and Women's Hospital. Kathmandu, Nepal from April 2021 to June 2021. Ethical approval from the institutional review board (IRB Ref. No. 854/2077/78) was taken before the study. Pregnant women beyond 34 weeks of gestations admitted in maternity hospital were included in the study. While the patients in labour, those with medical disorder of pregnancy, urinary tract infection on admission and those with history of urinary tract or pelvic surgery were excluded. Sample size was calculated by using the formula,

Sample size (n) = Z^2^ × p × q / e^2^

   = 1.96^2^ × (0.506 × 0.494) / (0.06)^2^

   = 268

Where,

n = sample size,
Z = 1.96 at 95% Confidence Interval.
p = prevalence of urinary incontinence taken from a previous study, 50.6%,^10^
q = 1-p,
e = margin of error, 6%

However we took a sample of 277. Convenience sampling method was used. The written consent of all the participating pregnant women was taken and the confidentiality was maintained throughout the study.

The study questionnaire consisted of four parts. The first included demographic variables like age, occupation, address, educational level, parity, mode of prior deliveries, weight gain, LUTS, pre-pregnancy UI and UTI. The second included the International Consultation on Incontinence Questionnaire-Urinary Incontinence-Short Form (ICIQ-UI-SF).^11^ The third included the Nepali version of ICIQ-UI-SF questionnaire and the fourth had the Consent. The Statistical Package for Social Sciences (SPSS) version 27 was used to collect and analyse the data. Point estimate at 95% Confidence Interval was calculated along with frequency and proportion for binary data.

## RESULTS

Out of 277 patients, urinary incontinence was present among 26 (9.4%) (95% Confidence Interval= 5.9612.84) of the pregnant women admitted in the third trimester of pregnancy. Stress urinary incontinence was the most common type present on 16 (61.6%) women, where leaks occurred during cough, sneeze and physical exertion while four (15.4%) had urge urinary incontinence and six (23%) had mixed urinary incontinence (Table 1).

**Table 1:** Showing types of urine leak.

When does urine leak	n (%)
Leaks before you get to toilet	4 (15.4)
Leaks when you cough or sneeze	12 (46.2)
Leaks when you are physically active/exercise	4 (15.4)
Leaks for no obvious reason and/or before you get to toilet and/or on cough or sneeze or on physically active/exercise	6 (23)

Majority 17 (65.4%) of women had 2-3 incontinent leaks a week and most of them 18 (69.3%) confirmed leaks in small amounts when ICIQ-UI-SF questionnaire was assessed (Table 2).

**Table 2:** Showing the severity (n = 26).

Severity	n (%)
Slight	10 (38.5)
Moderate	15 (57.7)
Severe	1 (3.8)
Very severe	0

This table also shows 10 (38.5%) patients had slight to 15 (57.7%) moderate impact of UI based on ICIQ-UI-SF score in range of 0-21 scale. One (3.8%) patient had significant overall interference with value more than five.

The average age of the population was found to be between 21-25 years 112 (40.4%) with majority of Tibeto-Burmese ethnicity 196 (70.8%). About 198 (71.5%) of the population were housewife with majority having secondary level education 135 (48.7%). One hundred seventy two (62.1%) of the pregnant women had weight gain between 10-12 kg (max. 18 kg and min. 7 kg). It shows majority 202 (72.9%) did not consume alcohol. Similarly, smoking was present among 39 (14.1%) and 103 (37.2%) drank tea with 23 (8.3%) drinking more than 3 cups per day. Constipation was seen in 95 (34.3%) of the women. The majority i.e. 246 (88.8%) had moderate physical activity and 3 (1.1%) had heavy physical activity. Majority 148 (53.4%) were multigravida, with 11 (3.9%) being gravida 4 or more and 129 (46.6%) were primigravida (Table 3).

**Table 3:** Socio-demographic and risk factors for urinary incontinence.

Age		n (%)
1	15-20	36 (13)
2	21-25	112 (40.4)
3	26-30	66 (23.8)
4	31-35	47 (17)
5	>36	16 (5.8)
Ethnicity		
1	Tibeto-Burmese	196 (70.8)
2	Indo-Aryans	81 (29.2)
Occupation		
1	House wife	198 (71.5)
2	Manual workers	20 (7.2)
3	Professional	53 (19.1)
4	Others	6 (2.2)
Education		
1	Uneducated	10 (3.5)
2	Primary level (Class 1-6)	64 (23.1)
3	Secondary level (Class 7-12)	135 (48.7)
4	Bachelor and above	68 (24.5)
Alcohol		
1	yes	75 (27.1)
2	no	202 (72.9)
1	yes	39 (14.1)
2	no	238 (85.9)
Tea		
1	1-2 cups	80 (28.9)
2	3 or more cups	23 (8.3)
3	no	174 (62.8)
Physical activity		
1	no	4 (1.4)
2	mild	24 (8.7)
3	moderate	246 (88.8)
4	heavy	3 (1.1)
Constipation		
1	yes	95 (34.3)
2	No	182 (65.7)
Gravidity		
1	Primi gravida	129 (46.6)
2	Gravida 2	96 (34.7)
3	Gravida 3	41 (14.8)
4	Gravida 4 and more	11 (3.9)
Weight Gain during current pregnancy		
1	7-9 kg	44 (15.9)
2	10-12 kg	172 (62.1)
3	13 or more kg	61 (22.0)

Majority 197 (71.2%) of the 277 pregnant women had features of lower urinary tract syndrome. Most of these women were having increased daytime frequency 172 (62%), and 10 (3.6%) had nocturia. Only 4 (1.4%) had urgency and one (0.4%) had hesitancy (Table 4).

**Table 4:** Lower Urinary Tract Symptoms.

Symptoms	n (%)
No symptoms	80 (28.8)
Increased day time frequency	172 (62.1)
Nocturia	10 (3.6)
Urgency	4 (1.4)
Hesitancy	1 (0.4)
Increased day time frequency + Nocturia	6 (2.2)
Increased day time frequency + Nocturia + Urgency	2 (0.7)
Nocturia + Hesitancy	1 (0.4)

## DISCUSSION

Among 277 of pregnant women under study, 26 women (9.4%) reported urinary incontinence even though 71.2% had lower urinary tract symptoms (LUTS). This finding is similar to the figures of 11.4% observed by Bekele, et al. in Ethiopia and 21% reported by Adaji, et al. in Nigeria.^12-13^ These prevalence differs to 42% average prevalence seen in a systemic review and meta-analysis of 44 studies.^2^ This variation could be due to the study population, operating definitions, trimester of pregnancy, parity and study methodology.^2^ Though definition of UI as any urine loss was taken, the low prevalence may be under reporting of UI as the questionnaires were filled up by patients themselves.

The difference in the prevalence may be because of the fact that UI may had caused minimum bother to them, or the idea that UI would improve by itself or wanting to postpone until after the delivery as observed by Moossdorff.^7^ As there was no follow up on UI in our study, it has been stated that introduction and follow up probe questions about UI results in doubling of the prevalence rate.^14^

The most common type of UI was stress urinary incontinence 61% followed by mixed urinary incontinence in 23% and Urgency urinary incontinence in 15%. The high prevalence of SUI followed by MUI and UUI has also been noted.^15-17^

The present study showed stress incontinence as the commonest followed by MUI and UUI. These findings are consistent with the study by Nigam, et al. and Abdullah, et al. who reported prevalence of stress urinary incontinence of 72.7% and 64.8% respectively.^15,16^

Most of the previous studies described advance age, increased BMI, smoking, constipation, alcohol, smoking, tea, and lack of activity were associated with risk of development of UI in pregnancy.^5,9,17-19^ Smoking, constipation, occupation and weight gain during pregnancy were significantly associated with UI in pregnancy. The possible explanation for smoking associated with UI are smoking associated cough, antiestrogenic effect on collagen of urethra and bladder neck and decreased smooth muscle tone.^20^ The incidence of urinary tract infection was 2.9% which is significantly low than that seen in high incidence of UI.^15^

Constipation was another significant factor associated with UI in pregnancy, which was also noted in other studies. Study by Bekele showed 12% increased change of UI if a women often experienced constipation.^12^ Similarly study by Dinç also showed increased prevalence of UI with constipation.^5^ This association could be explained by the resulting pelvic floor muscle weakness as a result of constipation.^12^

Similarly, the occupation was associated with UI in pregnancy. In our study, SUI was common among pregnant women who were housewife. The sudden urine leakage is a bothersome symptom which can diminish quality of life and disrupt the daily routine.^21^ Woman commonly assume a role of less physically demanding nature avoiding close working partners for fear of urine smell. This may be a coping strategy for them to curtail professional involvement which needs further research in our population.^22^

The 62.1% of the study population had weight gain of 10-12 kg during the pregnancy, which showed low statistical significance for UI. This could be due to the weakening of the pelvic floor muscles for developing UI due to low intake of protein.^23^ High weight gain during pregnancy is believed to contribute significantly to the increase of UI during and after pregnancy, but epidemiologic studies assessing this relation have been inconclusive.

Increased day time frequency is the most common physiology during the pregnancy. Our study reported 62% of frequency among the study subjects including 2.9% of women who had urinary tract infection. Similar findings were reported by Nigam, et al.^15^ Nocturia which is defined as waking to pass urine during the main sleep period seen in 3.6% of the study population, this is in contrast to high prevalence of 89% seen by Nigam, et al. where the majority of study population had UTI. The prevalence rate of nocturia may not vary only with the definition adopted but with the gestational age of pregnancy.^15^

ICIQ-UI-SF measures the UI in relation to frequency, amount, interference to everyday life and they add up to the ICIQ-UI-SF score, rating of UI symptoms in the past four weeks. It shows the severity of the urogenital complaints and high score showing the severity. The final self-diagnostic item helps us to differentiate the type of UI. The majority of the pregnant women had moderate (57.7%) to mild (38.5) severity which is similar to study by^7^ As the severe bother (>5) of UI to daily activity was only present in 3.8% of the study population may explain low reporting of UI and less concerned to report UI in pregnancy.

Even though significant percentage of women were having LUTS and UI, none had sought medical help. Pregnant women might be insufficiently aware that women with UI during pregnancy have a two to six fold risk of UI post-partum, depending upon the severity of UI in pregnancy and the immediate postpartum days.^22^ Thus, with high prevalence of LUTS reporting in contrast to low prevalence with UI, may show that the botherlessness of UI, which causes minimum discomfort to most pregnant women. This is consistent with higher level of bother and knowledge about information regarding availability of management of LUTS and UI being associated with high help seeking behaviour.^5,23^

Our study was limited to pregnant women admitted in a tertiary hospital after third trimester of pregnancy. The study period of three months duration might be missing those coming over other months. As the pregnant mothers were asked about urinary incontinence during final months, they may be anxious to reveal the data as they may consider it would impact or alter the management of their deliveries and the free safe motherhood service at the hospital.

UI and LUTS are common conditions during pregnancy, which carries a significant impact on quality of life. If all pregnant women are made aware of these conditions, as an integral part of antenatal check-up, all women who had any of these complaints would come forward for their management.

## CONCLUSIONS

Smoking, constipation, occupation and weight gain during the pregnancy were related to prevalence of urinary incontinence during the third trimester of pregnancy. Stress urinary incontinence was the most common form of urine incontinence with mild to moderate impact on their quality of life. Lower urinary tract symptoms are common with increased day time urine frequency being the most common symptoms. Addressing urinary incontinence from the first trimester would yield a positive response on urinary incontinence.
